# Multi-sensitization to hymenoptera venoms: diagnostic and clinical features

**DOI:** 10.1186/1939-4551-8-S1-A144

**Published:** 2015-04-08

**Authors:** Keity Souza Santos, Karine Marafigo De Amicis, Alexandra Sayuri Watanabe, Clóvis ES Galvão, Jorge Kalil, Fabio FM Castro, Mario Sergio Palma, Daniele Danella Figo, Jose Roberto Dos Santos Pinto

**Affiliations:** 1 University of Sao Paulo School of Medicine, FMUSP, Brazil; 2 Asbai, Brazil; 3 Clinics Hospital of FMUSP, Brazil; 4 Heart Institute (InCor), Brazil; 5 Universidade Estadual Paulista, Brazil

## Background

Double sensitization to both honeybee (*Apis mellifera*) and Yellow Jacket (*Vespula* ssp.) venom is common in up to 59% of Northern European Hymenoptera venom allergic patients and this rate is more than 50% in the United States. In Brazil yellow jacket is not a common wasp, but *Polistes* sp. and *Polybia paulista* poses the major risk for Brazilian patients. Reports about double sensitization involving honey bee and fire ant (*Solenopsis invicta*) are rare and there is nothing described about multi-sensitization to insects. Cross-reacting carbohydrate determinants (CCDs) are not present in *Polistessp.* venom and are not yet described for *Polybia paulista* neither *Solenopsis invicta*. Component-resolved analysis with recombinant species-specific major allergens may help to distinguish true double sensitization from cross-reactivity, except for *Polybia paulista*allergens for which these commercial tests are not yet available. Although there is no international consensus on whether immunotherapy regimens should generally include all venoms in multi-sensitized patients the recommendation is that immunotherapy (IT) should be extended to all venoms for which test results are positive and patients might potentially react to.

## Methods

We selected a group of ten patients with clinical manifestations of anaphylaxis presenting symptoms that included urticaria, angioedema, diarrhea, respiratory symptoms and loss of consciousness that are sensitized to honeybee, wasps (*Polistes* and *Polybia*) and fire ant. They were tested by ImmunoCap, Skin prick test (SPT), Dot Blot and Western Blotting (WB) with *Apis mellifera*, *Polistes* sp. and *Solenopsis invicta* extracts commercially available and also *Polybia paulista* venom extract produced in our laboratory.

## Results

Patients are positive to four venoms tested in Dot blot, WB and SPT. Five patients presented ImmunoCAP <0.35 for one or two venoms tested. WB revealed that patients are recognizing different bands in gel when comparing different venoms suggesting there is no cross-reactivity. Some bands recognized by specific IgEs would be new allergens, since they present distinct molecular weights from allergens already described. Cross-reactivity due to CCD recognition remains to be confirmed.

## Conclusions

This is the first report of multisensitization to honeybee, *Solenopsis*, *Polybia and Polistes* and considering clinical history, SPT and laboratory results patients presented here should be submitted to IT to all venoms tested. It is important to remark that *Polybiapaulista* venom is not commercially available for treatment and IT to Solenopsis is still not well established. Next steps are to check the presence of CCD in *Solenopsis* and *Polybia* venoms and also to identify new IgE reacting molecules.

